# Hematological and Genetic Predictors of Daytime Hemoglobin Saturation in Tanzanian Children with and without Sickle Cell Anemia

**DOI:** 10.1155/2013/472909

**Published:** 2013-04-03

**Authors:** Sharon E. Cox, Julie Makani, Charles R. Newton, Andrew M. Prentice, Fenella J. Kirkham

**Affiliations:** ^1^MRC International Nutrition Group, London School of Hygiene & Tropical Medicine, London WC1E 7HT, UK; ^2^Muhimbili Wellcome Programme, Muhimbili University of Health and Allied Sciences, Dar-es-Salaam, Tanzania; ^3^Nuffield Department of Clinical Medicine, University of Oxford, Oxford OX3 7BN, UK; ^4^Department of Psychiatry, University of Oxford, Oxford OX3 7JX, UK; ^5^University College London, Institute of Child Health, London WC1E 6BT, UK

## Abstract

Low hemoglobin oxygen saturation (SpO_2_) is common in Sickle Cell Anemia (SCA) and associated with complications including stroke, although determinants remain unknown. We investigated potential hematological, genetic, and nutritional predictors of daytime SpO_2_ in Tanzanian children with SCA and compared them with non-SCA controls. Steady-state resting pulse oximetry, full blood count, transferrin saturation, and clinical chemistry were measured. Median daytime SpO_2_ was 97% (IQ range 94–99%) in SCA (*N* = 458), lower (*P* < 0.0001) than non-SCA (median 99%, IQ range 98–100%; *N* = 394). Within SCA, associations with SpO_2_ were observed for hematological variables, transferrin saturation, body-mass-index *z*-score, hemoglobin F (HbF%), genotypes, and hemolytic markers; mean cell hemoglobin (MCH) explained most variability (*P* < 0.001, Adj *r* ^2^ = 0.09). In non-SCA only age correlated with SpO_2_. *α*-thalassemia 3.7 deletion highly correlated with decreased MCH (Pearson correlation coefficient −0.60, *P* < 0.0001). In multivariable models, lower SpO_2_ correlated with higher MCH (*β*-coefficient −0.32, *P* < 0.001) or with decreased copies of *α*-thalassemia 3.7 deletion (*β*-coefficient 1.1, *P* < 0.001), and independently in both models with lower HbF% (*β*-coefficient 0.15, *P* < 0.001) and Glucose-6-Phosphate Dehydrogenase genotype (*β*-coefficient −1.12, *P* = 0.012). This study provides evidence to support the hypothesis that effects on red cell rheology are important in determining SpO_2_ in children with SCA. Potential mechanisms and implications are discussed.

## 1. Introduction

Hemoglobin oxygen desaturation in the absence of acute illness is common in children with Sickle Cell Anemia (SCA), and is associated with higher cerebral blood flow velocities [1, 2], and with risk of complications including stroke [3]. The underlying mechanisms of hemoglobin oxygen desaturation in SCA are poorly understood but may involve the severity of anemia [4] as well as differences in hemoglobin oxygen affinity compared to hemoglobin A (HbA), with increased expression of 2.3 DPG in hemoglobin S (HbS) resulting in a right-shifted hemoglobin oxygen affinity curve and other differences in red cell physiology [5]. Other potential causes include a history of acute chest syndrome and reduced pulmonary [6] and cardiac function [7]. Coinheritance of alpha-thalassemia deletions and glucose-6-phosphate deficiency (G6PD) may affect the degree of anemia [8, 9] whilst alpha-thalassemia status modifies red cell indices [10–12] and rheology [13], as can iron status [14]. We therefore investigated potential hematological, genetic, and nutritional predictors of daytime hemoglobin oxygen saturation in Tanzanian pediatric patients homozygous for HbS (SCA) and in non-SCA local controls.

## 2. Patients **a**nd Methods

Ethical permission was granted by the Muhimbili University of Health and Allied Sciences Ethics Committee (MU/RP/AECNoI.XII/77). Written informed consent was obtained from parents or guardians in their own language. 

### 2.1. Patients and Clinical Procedures

Children (less than 17 years) with SCA (HbSS genotype) were enrolled in the SCD cohort study at Muhimbili National Hospital, Dar-es-Salaam [15]. Resting pulse oximetry data (Masimo Radical, Masimo Corporation, USA) and blood samples were collected at routine outpatient clinic visits between November 2007 and December 2008. Analysis was limited to data collected at a single steady-state time point. A strict definition of steady state was employed (temperature <37.5°C, no malaria parasitaemia, no reported pain, no blood transfusion within 90 days or hospital admission within 30 days on either side of the selected time point) and determined to be clinically well by the attending doctor. All cohort children are routinely prescribed folate supplementation (5 mg/day). 

Non-SCA children were those who presented for sickle testing between October 2004 and December 2008 but who had HbAA or HbAS by hemoglobin electrophoresis. None of the children had malaria parasitaemia or fever (temperature >37.4°C) and all were clinically well.

### 2.2. Laboratory Procedures

Blood samples were collected between 8 and 10 am. Full blood counts were performed using an automated cell counter (Pentra 60, Horiba ABX, Kyoto, Japan). Serum iron and total iron binding capacity were measured in serum samples stored at −80°C (Architect C8000, Abbott, New York, USA). Transferrin saturation was calculated from serum iron and total iron binding capacity. Lactate dehydrogenase (LDH) and bilirubin (total and conjugated) were measured in fresh serum samples (Architect C8000, Abbott, New York) by Muhimbili Central Pathology Laboratory. 

Children attending screening for sickle status were typed for HbS by alkaline Hb electrophoresis (Helena, Sunderland, Tyne and Wear, UK). Children enrolled in the Muhimbili Sickle cohort also had hemoglobin fractions, including HbF, quantified by HPLC using the *β*-thalassemia Short Programme on the Variant analyzer (BioRad, Hercules, CA, USA). In addition, children enrolled in the Muhimbili Sickle Cohort had HbSS status confirmed by genotype and were genotyped for the 3.7 alpha-thalassemia deletion using a PCR-based method and agarose gel visualization as per published methods [16] and the 202- and 376-single nucleotide polymorphisms (SNPs) for glucose 6-phosphate dehydrogenase deficiency (G6PD) and HbS using multiplex Sequenom [17]. 

### 2.3. Data Analysis

Data were analyzed using STATA 11-IC (StataCorp, College Station, TX, USA). Daytime SpO_2_ is not normally distributed due to an excess of observations having the maximum possible value of 100% and a skewed distribution towards the lower values. The data are not normalized by the usual log transformations. Thus we investigated the use of negative binomial regression of count data using a new variable of SpO_2_-100 compared to zero-inflated negative binomial regression. Vuong tests [18] indicated no strong consistent preference for either models across the explanatory variables tested. As we had no prior hypotheses that mechanisms to predict 100% versus <100% SpO_2_ may differ from mechanisms underlying the degree of desaturation, we selected the negative binomial regression model. We next compared the results of these models to those from simple linear regression of the nontransformed data and observed no major differences in the results. Whilst analysis of the residuals from the linear regression models indicated some skew, this was not judged to be sufficient to render the results invalid, which were in agreement with those from the better fitting negative binomial regression models. Thus for ease of presentation and interpretation the results of the linear regression models are presented. *P* values <0.05 were considered significant. 

## 3. Results 

**Figure 1:**
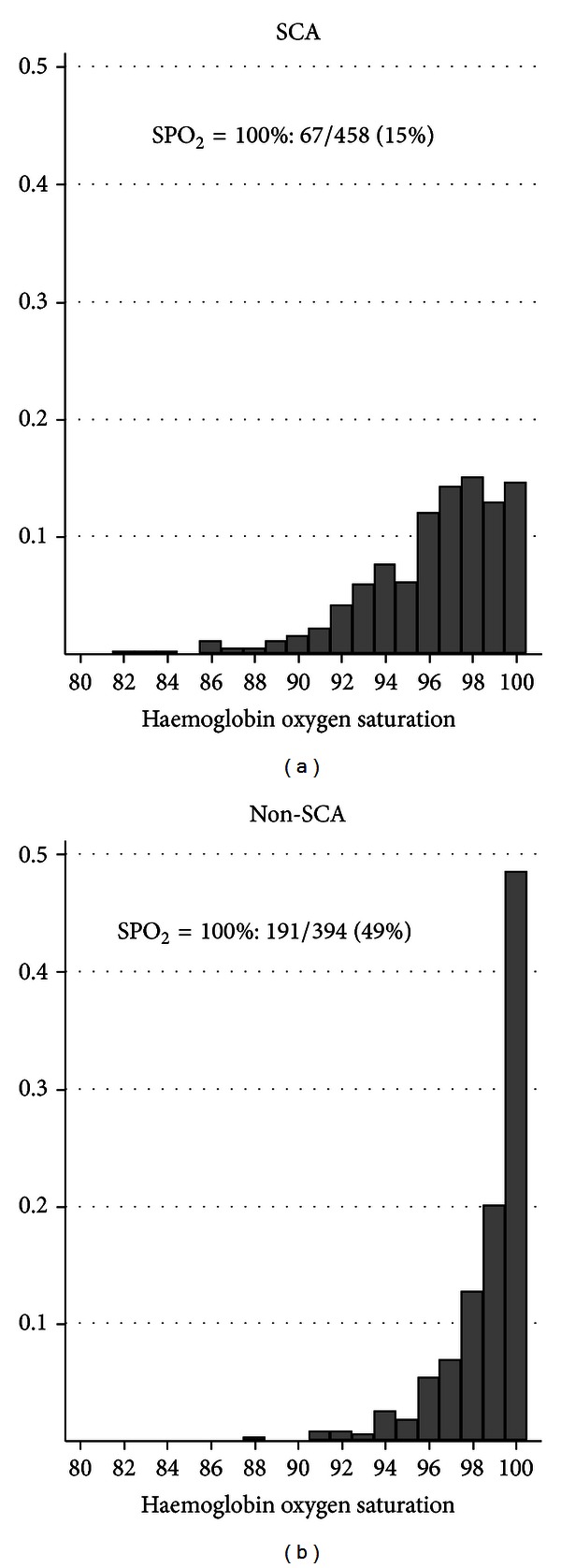
Distribution of daytime hemoglobin oxygen saturation in children with SCA compared to non-SCA.

Complete hematological, genetic, and iron status (transferrin saturation) data were available for 458 SCA children. None of the children were receiving hydroxyurea or routine blood transfusions. None of the children had received more than 4 blood transfusions in their lifetime. In addition, complete hematological and pulse oximetry data were available for 394 non-SCA children, although transferrin saturation data were only available in a small subset of 62 children and genotyping was not conducted in these controls. Pulse oximetry, hematological and iron status data for the two groups of children are summarized in Table 1. Of the 458 SCA children, 239 were boys (52%) with a mean age of 9.7 y (SD 4.3 y) compared to 212 boys (54%) in the 394 non-SCA children with a mean age 7.0 y (SD 4.7 y). In the SCA children median daytime SpO_2_ was 97% (IQ range 94–99%) (Table 1), significantly lower (Wilcoxon rank sum test; *P* < 0.0001) than in the non-SCA children (median 99%; IQ range 98–100%) (Table 1 and Figure 1). Hemoglobin concentration, red cell indices (red blood cell count (RBC), mean cell hemoglobin (MCH), and mean cell hemoglobin concentration (MCHC), mean cell volume (MCV)), and markers of hemolysis (LDH and unconjugated bilirubin) were significantly different between the SCA and non-SCA groups (Table 1). The proportion of SCA children with low transferrin saturation (<16%) indicating probable iron deficiency, was 28% (126/458) but was lower than that in non-SCA Tanzanian control children (32/62, 52%) (Table 1). Only 9 (2%) of the SCA and none of the non-SCA patients had high transferrin saturation (>55%) which might indicate iron over-load. Forty-one percent of the SCA children (192/458) were heterozygous for the 3.7 alpha-thalassemia deletion and 17% were homozygous (78/458). Eleven percent of SCA children were heterozygous females for both the −202 and −376 G6PD single nucleotide polymorphisms (SNPs) which results in a moderate phenotype [19], whilst a further 12% (54/458) of SCA children were affected homozygous females or affected males.

Predictors of SpO_2_ in SCA are shown in Table 2. Within the SCA population all of the hematological variables were significantly associated with SpO_2_. Higher hemoglobin, RBC, and HbF% were associated with greater SpO_2_, whilst higher MCH, MCHC, and MCV were associated with lower SpO_2_. MCH was the single biggest contributor to the variation in SpO_2_ as indicated by the still modest adjusted *r* ^2^ value of 0.09. There was weak evidence of a negative association between transferrin saturation and SpO_2_. In the non-SCA population the same hematological variables were tested for associations with SpO_2_, as well as Hb phenotype, AS versus AA. However, the only predictor of SpO_2_ in this group was a positive association for age (beta coefficient 0.05, *P* = 0.024) with no evidence to suggest even weak effects of any of the hematological indices. 

In the SCA population for whom genotypes were available, coinheritance of the alpha-thalassemia 3.7 genotype was associated with increased SpO_2_, whilst co-inheritance of G6PD deficiency was associated with decreased SpO_2_. 

Pair-wise associations between the different explanatory variables for daytime SpO_2_ within the SCA group were investigated and are presented in full in Supplementary Table 1  available online at http://dx.doi.org/10.1155/2013/472909. Alpha-thalassemia genotype was highly associated with all of the hematological variables, with the number of 3.7 deletions being associated with higher hemoglobin (Pearson correlation coefficient 0.23); RBC count (0.55); lower MCV (−0.24), MCHC (−0.32), and MCH (−0.60); and also lower hemolytic markers, particularly unconjugated bilirubin (−0.26), all with *P* values <0.0001. Interestingly, presence of the 3.7 alpha-thalassemia deletion was also associated with improved nutritional status as determined by body-mass-index *z*-score. Higher MCH and MCV were associated with lower hemoglobin in the SCA population whilst HbF% was associated with higher hemoglobin. Transferrin saturation was positively associated with hemoglobin (0.17) and also higher MCH (0.33), both *P* values <0.0005, but was not associated with MCV. Interestingly, G6PD genotype was not significantly associated with any of the other variables.

Correlations between the hematological variables were similar in the non-SCA population compared to SCA (data not shown) except that higher MCV was correlated with higher hemoglobin (0.64, *P* < 0.001) but was not associated with RBC count and no association was apparent between hemoglobin and MCHC.

In multivariable models predicting SpO_2_, the inclusion of MCH with alpha-thalassemia genotype resulted in a diminished and nonsignificant association for alpha-thalassemia. However, in models including either MCH or alpha-thalassemia there were an independent effect of HbF% and weak evidence of an independent effect of G6PD limited to affected males and homozygote females (Supplementary Table 2), but with the greatest variation explained by a model including MCH and HbF% (adjusted *r* ^2^ = 0.15, *P* < 0.001, *N* = 315). 

## 4. Discussion

Few studies have attempted to determine the hematological or genetic correlates of daytime SpO_2_. In this study we report that higher daytime SpO_2_ in children with SCA is associated with higher red cell count and hemoglobin, but with lower MCH or MCHC. Coinheritance of the alpha-thalassemia 3.7 deletion in our SCA population is strongly associated with increased hemoglobin, red cell count and decreased MCH and MCHC and also with increased SpO_2_, but not independently of these factors. Thus the basis of the association between alpha-thalassemia genotype and SpO_2_ would appear to be via its effects on red cell indices. In Kenyan nonsickle populations, alpha-thalassemia 3.7 deletion copy number is also associated with slightly increased hemoglobin levels and increased red cell counts, but effects on anaemia appear to be modulated by sickle trait status [20]. This suggests that the decreased anemia and increased red cells observed in SCA plus alpha-thalassemia results from decreased rate of red cell destruction, proposed to be due to lower HbS concentration in red cells, and thus reduced HbS polymerization which is concentration dependent as well as being increased by desaturation [11]. This is supported by the observation that alpha thalassemia was associated with lower levels of LDH and unconjugated bilirubin as markers of hemolysis. Red cell count and hemoglobin were not associated with SpO_2_ when adjusting for MCH and thus anemia does not appear to be a direct cause of low SpO_2_ in our population. A beneficial effect of alpha thalassemia on daytime SpO_2_ has also been observed in Jamaican children with SCA, [21] but in contrast to our study, there were also independent effects of hemoglobin, whilst neither MCH, or MCHC was associated with SpO_2_ [21].

It is interesting to note that within the non-SCA population none of the tested variables were associated with SpO_2_. This may have been the result of the reduced variability in SpO_2_ in the non-SCA population, or reflect sickle-specific effects of red cell indices on SpO_2_. Again, this contrasts with observations in older Jamaican children who were HbAA (15–18 years) in whom hemoglobin and MCV (but not MCH or MCHC) were associated with SpO_2_. 

Our observation of a beneficial effect of co-inheritance of alpha-thalassemia on daytime SpO_2_ in children with SCA appears to be in direct contrast to our previous observation of a negative effect on mean overnight SpO_2_, measured in a small group (*N* = 30) of similar aged Tanzanian children with SCA [22]. However, this effect of alpha-thalassemia appeared to be confounded by the strong negative association between transferrin saturation and mean overnight SpO_2_. We concluded that this association was most likely the result of reverse causality from nocturnal chronic and intermittent hemoglobin desaturation causing increased transferrin saturation due to upregulation of hypoxia-inducible factor, which has downstream effects of increasing iron absorption, which may also be increased by alpha-thalassemia [20]. Thus we suggest that in a larger dataset, alpha-thalassemia may also have a beneficial effect on mean overnight SpO_2_. 

Similar to Jamaican [21] and American SCA children [6], HbF% was positively and independently associated with SpO_2_. It is not possible in the current study to determine if an effect of HbF% is via direct effects on increased hemoglobin oxygen affinity or through indirect effects on rates of sickling or red cell rheology. Interestingly alpha-thalassemia was also associated with increased HbF%, perhaps through increased rate of release of immature red cells from ineffective erythropoiesis. The co-inheritance of G6PD deficiency was negatively associated with SpO_2_. As there was no evidence of an association between G6PD and levels of hemolytic markers, as has been observed previously in SCA [9], the effect on SpO_2_ is likely through another mechanism. 

Lower daytime and nocturnal SpO_2_ in SCA are associated with poor clinical outcomes. This includes elevated cerebral blood flow velocities [1, 2] and an increased risk of stroke [3, 23]. Co-inheritance of alpha-thalassemia is protective against elevated cerebral blood flow velocities in children with SCA [24, 25] and is associated with reduced stroke risk [26], whilst G6PD increases the risk [24]. Thus the mechanisms behind these associations may, at least in part, be through effects on hemoglobin oxygen saturation and subsequent endothelial dysfunction [27, 28] as well as other potential effects of red cell deformability and endothelial adhesion [24]. 

In conclusion, in our population of Tanzanian children with SCA, the strongest predictor of lower SpO_2_ is increased mean cell hemoglobin, which is strongly correlated with a decreased copy number of the 3.7 alpha-thalassemia deletion. The degree of anemia, red cell count, and hemolytic markers do not independently correlate with SpO_2_, thus suggesting that effects of mean cell hemoglobin and the alpha-thalassemia genotype on SpO_2_ are mediated through effects on red cell rheology. 

## Supplementary Material

Table 1 of the supplementary material contains the results of investigating the associations between the exposure variables in order to assess for potential confounding or co-linearity before including variables in multivariable models or to consider possible mechanisms of action. Table 2 reports four different possible multivariable models of independent predictors of daytime SpO2 in SCA with either the alpha-thalassemia 3.7 deletion or mean cell haemoglobin plus G6PD genotype or HbF%.

## Figures and Tables

**Table 1:** Daytime pulse oximetry data, haematological and haemolytic indices and iron status in Tanzanian SCA and non-SCA children.

Variable	SCA children with daytime oximetry (N = 458)	Non-SCA children with daytime oximetry (N = 394)
Pulse oximetry		
Median daytime SpO_2_ (%)	97 (IQ range 94–99)	99 (IQ range 98–100)***
Haematology		
Mean hemoglobin* (g/dL)	7.4 (SD 1.17)	10.9 (SD 2.2)***
Mean red cell count × 10^−9^	2.89 (SD 0.66)	4.71 (SD 0.86)***
Mean cell hemoglobin concentration (g/dL)	31.9 (SD 1.52)	32.3 (SD 1.60)**
Mean cell hemoglobin (pg/cell)	26.1 (SD 3.31)	23.6 (SD 3.86)***
Mean cell volume* (fL)	79.5 (SD 15.28)	73.1 (SD 9.21)***
Median reticulocyte % (N = 440/335)	12.8 (IQ range 9.1–16.4)	2.4 (IQ range 0.8–6.7)***
Median hemoglobin F (Hb%) (*N* = 316/60)^†^	4.7 (IQ range 2.8–7.9)	0.6 (IQ range 0.2–1.0)***
Haemolytic markers		
Median lactate dehydrogenase (IU) (N = 427/340)	762 (533–1121)	498 (367–671)***
Median unconjugated bilirubin (μmol/L) (N = 417/181)	33.3 (IQ range 18.2–61.1)	7.2 (IQ range 3.5–12.5)***
Iron status		
Median transferrin saturation (%) (N = 458/62)	20.9 (IQ range 15.4–27.7)	14.9 (IQ range 9.1–25.3)**
Median serum iron (μmol/L) (N = 458/62)	11.5 (SD 5.7) (range 8.6–14.9)	10.0 (SD 8.5) (range 5.8–17.2)
Mean serum TIBC (μmol/L) (N = 458/62)	55.3 (SD 10.9) (range 47.7–62.75)	70.5 (SD 22.3) (range 50.4–84.3)***
Iron deficiency transferrin saturation < 16%	126/458 (27.5%)	32/62 (51.6%)***

^†^Within the SCA children, HbF% was not assessed at the same time point as the hematology and SpO_2_ data and was limited to children older than 5 years at the time of measurement of HbF% & SpO_2_. HbF% was available for some non-SCA children and was measured at the same time point as the SpO_2_.

*P < 0.05, **P < 0.01, ***P < 0.001.

**Table 2:** Predictors of daytime SpO_2_ in SCA.

Explanatory variable	β-coefficient	*P* value	Adj *r* ^2^
Hematological indices			
Hemoglobin (g/dL)	0.35	0.007	0.014
RBC × 10^−9^	1.10	<0.001	0.050
MCHC (g/dL)	−0.57	<0.001	0.073
MCH	−0.29	<0.001	0.090
MCV (fl)	−0.02	0.041	0.007
HbF% (*N* = 315)^†^	0.12	<0.001	0.026
Iron status and nutritional status			
Transferrin saturation %	−0.03	0.03	0.008
BMI Z-score (*N* = 451)	0.18	0.049	0.006
Hemolytic markers			
Lactate dehydrogenase (IU) {natural log} (*N* = 427)	−0.72	0.011	0.013
Unconjugated bilirubin (*μ*mol/L) {natural log} (*N* = 415)	−0.34	0.029	0.009
*α*-thalassemia genotype			
3.7 heterozygote versus Wild Type	1.29	<0.001	
3.7 homozygote versus Wild Type	2.04	<0.001	0.056*
G6PD genotype			
Heterozygote females versus Wild Type	−0.52	0.276	
Homozygote females and affected males versus Wild Type	−1.30	0.005	0.014*

*N* = 458 unless otherwise stated. *Adj *r* ^2^ for whole model. ^†^Analysis of SpO_2_ limited to those with HbF and SpO_2_ measured when patients were 5 years or older.
