# Supplementary material for: Hematological and Genetic Predictors of Daytime Hemoglobin Saturation in Tanzanian Children with and without Sickle Cell Anemia
Source: ISRN Hematol. 2013 Apr 3;2013:472909. doi: 10.1155/2013/472909 (PMC3649307; doi:10.1155/2013/472909)
Supplement: Supplementary file 1 — Table 1 of the supplementary material contains the results of investigating the associations between the exposure variables in order to assess for potential confounding or co-linearity before including variables in multivariable models or to consider possible mechanisms of action. Table 2 reports four different possible multivariable models of independent predictors of daytime SpO2 in SCA with either the alpha-thalassemia 3.7 deletion or mean cell haemoglobin plus G6PD genotype or HbF%. [file 472909.f1.docx]

**Table S1** Associations between the explanatory variables using Pearson correlation coefficients in SCA

|  | Hgb | RBC | MCV | MCHC | MCH | TS % | LDH | Uncon-jugated bilirubin | Age | Sex | BMI Z-score | Alpha-thal | G6PD | HbF% |
| --- | --- | --- | --- | --- | --- | --- | --- | --- | --- | --- | --- | --- | --- | --- |
| Hgb | --- |  |  |  |  |  |  |  |  |  |  |  |  |  |
| RBC | 0.72*** [457] | --- |  |  |  |  |  |  |  |  |  |  |  |  |
| MCV | -0.13** [457] | -0.42*** [457] | --- |  |  |  |  |  |  |  |  |  |  |  |
| MCHC | 0.20*** [458] |  |  | --- |  |  |  |  |  |  |  |  |  |  |
| MCH | -0.09^ [458] | -0.65*** [457] | 0.46*** [457] | 0.54*** [457] | --- |  |  |  |  |  |  |  |  |  |
| TS% | 0.17*** [458] |  |  | 0.19*** [458] | 0.24*** [458] | --- |  |  |  |  |  |  |  |  |
| LDH | -0.23***  [427] | -0.17***  [426] |  |  | 0.09^  [427] |  | --- |  |  |  |  |  |  |  |
| Uncon-jugated  bilirubin |  | -0.23***  [N=416] |  | 0.22***  [417] | 0.33**  [417] | 0.13**  [417] |  | --- |  |  |  |  |  |  |
| Age | 0.13** [458] |  |  | 0.39*** [458] | 0.15** [458] | 0.11* [458] | -0.14**  [427] | 0.17***  [417] | --- |  |  |  |  |  |
| Sex |  |  |  |  | -0.08^ [458] | -0.13** [458] |  |  |  | --- |  |  |  |  |
| BMI Z-score |  |  |  | 0.12* [451] |  |  | -0.15**  [N=420] | 0.10*  [410] | 0.31*** [451] | -0.24*** [451] | --- |  |  |  |
| Alpha-thal | 0.23*** [458] | 0.52*** [457] | -0.24*** [457] | -0.32*** [458] | -0.60*** [458] | 0.09^ [458] | -0.10*  [N=427] | -0.26***  [417] |  |  | 0.11* [451] | --- |  |  |
| G6PD |  | -0.09^ [457] |  |  |  | 0.08^ [458] |  |  |  | 0.08^ [458] |  |  | --- |  |
| HbF% | 0.17*** [448] |  | 0.08^ [447] | -0.11* [448] | 0.09^ [448] |  |  |  | -0.29*** [448] |  |  |  | -0.10* [448] | --- |

*** P<0.001; **P<0.01; *P<0.05; ^P<0.10

**Table S2**. Multivariable models for Daytime Hemoglobin Oxygen Saturation

| **Explanatory variable** | **Β-coefficient** | | **P-value** |
| --- | --- | --- | --- |
| ***Model 1 [N=319] Adjr^2^=0.070*** |  |  | |
| Number of 3.7 alpha-thalassemia deletions | 0.88 | <0.001 | |
| Hemoglobin F% | 0.13 | 0.003 | |
| ***Model 2 [N=319] Adjr^2^=0.132*** |  |  | |
| Mean Cell Hemoglobin | -0.32 | <0.001 | |
| Hemoglobin F% | 0.15 | <0.001 | |
| ***Model 3 [N=458] Adjr^2^=0.069*** |  |  | |
| Number of 3.7 alpha-thalassemia deletions | 1.05 | <0.001 | |
| G6PD Heterozygote females vs. Wild Type | -0.71 | 0.128 | |
| G6PD Homozygote females & affected males vs. Wild Type | -1.12 | 0.013 | |
| ***Model 4 [N=458] Adjr^2^=0.099*** |  |  | |
| Mean Cell Hemoglobin | -0.29 | <0.001 | |
| G6PD Heterozygote females *vs.* WT | -0.51 | 0.266 | |
| G6PD Homozygote females & affected males *vs.* WT | -1.12 | 0.012 | |
